# Pharmacogenomic of LH and its receptor: are we ready for clinical practice?

**DOI:** 10.1186/s12958-025-01359-2

**Published:** 2025-02-25

**Authors:** Alessandro Conforti, Raffaella Di Girolamo, Maurizio Guida, Carlo Alviggi, Livio Casarini

**Affiliations:** 1 Department of Neuroscience, Reproductive Science and Odontostomatology, University of Naples Federico II, Via Sergio Pansini, 5, Napoli, 80131 Italy; 2 Department of Public Health, University of Naples Federico II, Naples, Italy; 3 Unit of Endocrinology, Department of Biomedical, Metabolic and Neural Sciences, University of Modena and Reggio Emilia, Modena, Italy; 4 Center for Genomic Research, University of Modena and Reggio Emilia, Modena, Italy

**Keywords:** LH, LHCGR, Genetic variants, Pharmacogenomic, IVF, Ovarian stimulation

## Abstract

Luteinizing hormone (LH) is fundamental to support development and reproduction. It acts through a receptor expressed in the gonads, modulating mitogenic, anti-apoptotic, and steroidogenic signals. LH is also marketed as a drug for controlled ovarian stimulation (COS), where it is administered to women to support the action of follicle-stimulating hormone and can lead to specific responses, depending on the individual genetic background. These concepts underline the relevance of a pharmacogenetic approach to COS, in the attempt to optimize clinical outcomes and avoid adverse events. However, knowledge is currently limited by the paucity of clinical studies. This review aims to provide a comprehensive overview of LH and its receptor activity, starting from the description of their molecular pathways from in vitro studies. Data on LH action from in vivo studies were described, as well as the impact of LH and LH/choriogonadotropin (hCG) receptor genetic variants on folliculogenesis and its association with infertility or polycystic ovarian syndrome. Finally, evidence from clinical studies evaluating genetic polymorphisms in the context of assisted reproductive technology treatments and its implications for a pharmacogenomic approach were discussed.

**Supplementary Information:**

The online version contains supplementary material available at 10.1186/s12958-025-01359-2.

## Introduction

Luteinizing hormone (LH) is a gonadotropin fundamental to development and reproduction. It is a dimeric glycoprotein released in a pulsatile fashion by the pituitary gland and acts through the LH/choriogonadotropin (hCG) receptor (LHCGR), which is expressed in the gonads [1]. LH has a β subunit (LHβ), specific for receptor binding, and an α subunit shared with other structurally similar glycoproteins: follicle-stimulating hormone (FSH), thyroid-stimulating hormone (TSH), and hCG [2]. The β-subunit is coded by the *LHB* gene located in the genomic locus 19q13.33, while the α-subunit is coded by the *CGA* gene, located at 6q14.3, and is assembled with LHβ within pituitary gonadotropic cells. The final product is a ~ 33 kDa molecule that, in women of fertile age, exerts a key role in supporting the production of ovarian sex steroids and modulating mitogenic and anti-apoptotic signals. These functions sustain follicular growth, oocyte maturation, and ovulation, as well as the luteinization of granulosa cells.

LHCGR is coded by the homonym gene, located in the genomic position 2p16.3, which spans about 80 kbases with 11 exons and 10 introns, and consists in a common receptor for both LH and hCG [3]. These two ligands have different physiological functions: hCG is the pregnancy hormone, produced by trophoblast cells to induce progesterone production; hCGβ molecules are coded by cluster genes (*CGB*s), located in proximity of *LHB* and likely evolved from a common ancestral sequence [4]. However, hCGβ has an additional carboxyl-terminal peptide of about 30 amino acids carrying six glycosylation sites, and an extended half-life [5].

Although LH and hCG display these physiological, structural, and biochemical differences, they are marketed as recombinant or extractive and highly purified drugs to support FSH in controlled ovarian stimulation (COS), in the context of assisted reproduction [6]. The action of these molecules is modulated by hormone and receptor polymorphic variants that influence individual response to COS, as well as predisposition to diseases and adverse events [7]. The pharmacogenomic approach could represent an opportunity to improve the efficiency of COS [8]. Apart from FSH, it is widely acknowledged that LH is crucial for folliculogenesis [9, 10]. Several studies have demonstrated that a specific subset of women undergoing IVF treatment – namely, those with low prognosis according to POSEIDON criteria – may benefit from LH during COS [11–13]. Moreover, there are specific genetic variants of LH and its receptor that could benefit from FSH or LH dose adjustment during assisted reproductive therapy (ART) [14–16]. These concepts form the basis of the pharmacogenetic approach to assisted reproduction; this review provides a summary of the molecular pathways and pharmacogenetics of LH in clinical practice. Moreover, results from in vivo and in vitro studies about LH signaling, LHβ and LHCGR variants, as well as their clinical impact, were discussed.

## Materials and methods

We performed a literature search in PubMed, Scopus, Embase, and the ISI Web of Science database. The search terms were: ‘LHCGR’, ‘LH’, ‘polymorphisms’, ‘genetic variants’, ‘ART’, ‘IVF’, ‘polycystic ovarian syndrome’, and ‘PCOS’ from the inception to January 2024. The most relevant studies that analyzed the impact of LH or LHCGR polymorphism on IVF outcomes are summarized in Supplemental Table 1. No language or time restriction was adopted.

## Results

### Molecular pathways involved in LH signaling results from in vitro studies

LHCGR is a 7-transmembrane, class A, G protein-coupled receptor (GPCR) [1]; its active and inactive conformations have been recently resolved by cryogenic electron microscopy [17]. Hormone binding to the receptor extracellular domain induces a ‘push/pull’ movement involving the hinge region and impacting the spatial arrangement of the transmembrane stretches [17], resulting in the activation of multiple intracellular signaling cascades [3]. It is generally accepted that LHCGR mediates both cAMP and intracellular Ca^2+^ increase, as well as sex steroid hormone production. These molecules were detectable using the first, pioneering assays available, such as radioimmunoassay, and have long been considered the main players involved in LH/hCG signaling [18, 19].

cAMP and Ca^2+^ increase rapidly and belong to two separate signaling pathways mediated by different G proteins: G_α_s and G_α_q, respectively [20]. cAMP is a second messenger inducing the activation of PKA and the phosphorylation of CREB, before being metabolized to AMP [21]. In granulosa cells, relatively high intracellular cAMP concentrations have been linked to pro-apoptotic effects [22–24] and, at the same time, to the compartmentalization of progesterone synthesis and androgen conversion to estrogens [25].

**Fig. 1:**
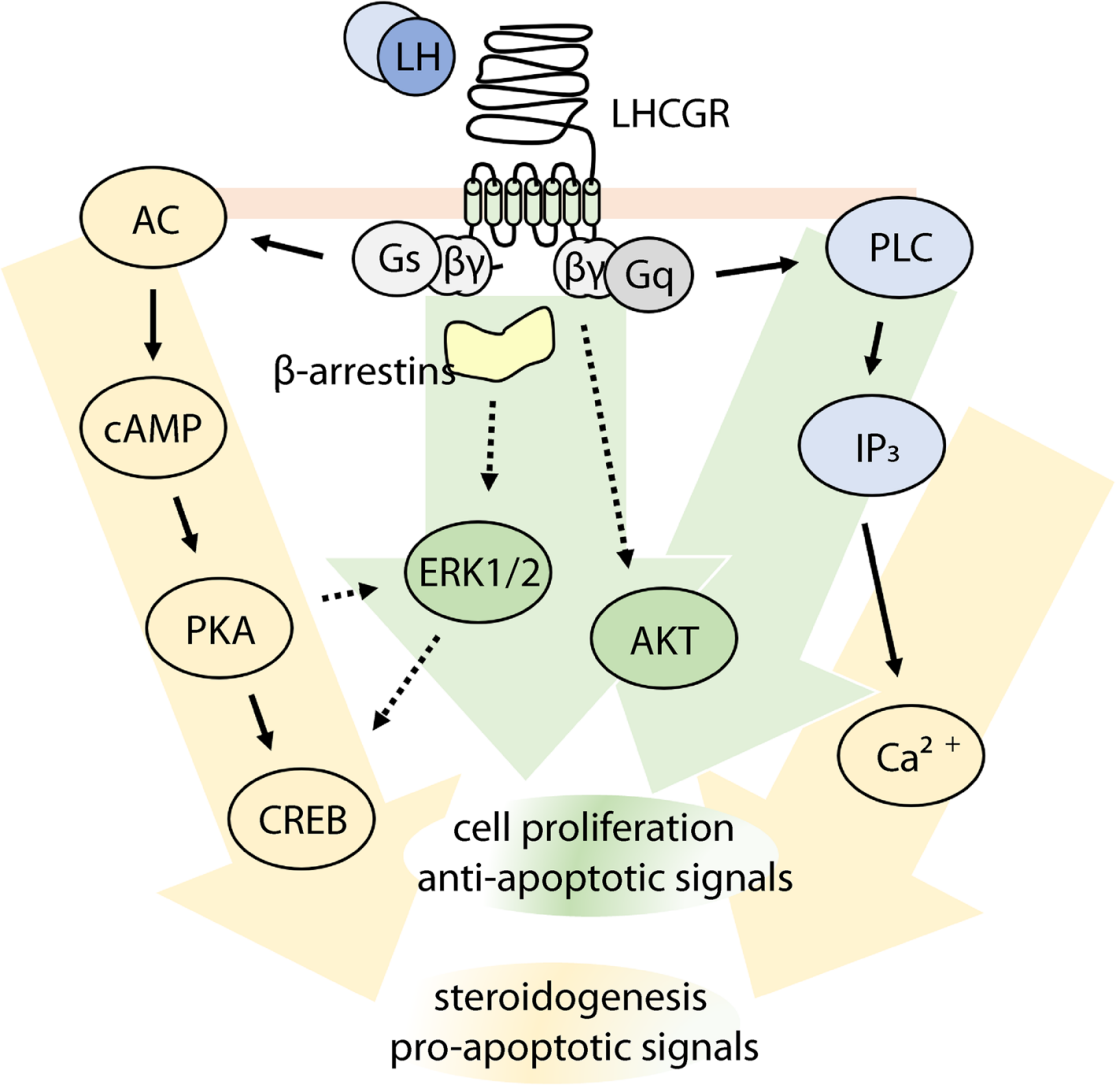
Main LHCGR-mediated signaling pathways. Upon hormone binding, LHCGR activates multiple signaling pathways via G protein and B-arrestin recruitment. These signaling cascades converge mainly into steroidogenic and pro-apoptotic stimuli, counterbalanced by mitogenic and anti-apoptotic signals

Androgens, mainly androstenedione, are produced by theca cells upon binding of phosphorylated CREB (pCREB) to *CRE* DNA target sequences. This is a PKA-dependent event that induces the transcription of steroidogenic enzyme-coding genes such as steroidogenic acute regulatory protein (*STARD1*), cytochrome P450 family 17 subfamily A member 1 (*CYP17A1*), and aromatase (*CYP19A1*) [26]. PKA activation is also followed by phosphorylation of the extracellular-regulated kinase 1/2 (ERK1/2), accompanying the inhibition of progesterone production and stimulation of androgens synthesis [27], and upregulating mitogenic processes in gonadal steroidogenic cells [28, 29]. Moreover, ERK1/2 phosphorylation is linked to the downregulation of receptor mRNA transcripts [28] and activation of GPCR kinases responsible for receptor phosphorylation and internalization into intracellular vesicles [29]. In particular, the compartmentalization of LHCGR is mediated by β-arrestins, which are proteins responsible for a second wave of ERK1/2 phosphorylation [30], occurring possibly as an opposing effect to cAMP-dependent pro-apoptotic signals [31] (Fig. 1).

In vitro experiments in transfected cells, expressing the murine receptor, demonstrated that gonadotropins are responsible for G_α_q protein and phospholipase C (PLC) activation, inositol trisphosphate (IP_3_) binding to calcium channels of endoplasmic reticulum, and mobilization of intracellular Ca^2+^ [20]. Calcium ions modulate the activity of protein calmodulin kinases, a key event to control cell proliferation [32] and transport of cholesterol into the mitochondrion, as a rate-limiting step for steroidogenesis [33, 34]. Interestingly, the half-maximal (EC_50_) hCG concentration activating the cAMP/PKA pathway is ~ 20 times lower than that required to trigger PLC/Ca^2+^ pathway activation, suggesting that LHCGR has dual signaling potential [35].

Recent scientific advancements have demonstrated the existence of multiple intracellular signaling cascades, providing a more detailed picture of LH- and hCG-mediated signals. For instance, the βγ dimer of G protein may indirectly lead to protein kinase B (AKT) activation, which upregulates survival signals [36], inhibits aromatase [37] and supports *STARD1* expression [38]. The preferential activation of specific signaling patterns depends on several factors. First, LHCGR signaling may be allosterically modulated by other 7-transmembrane partners, which can physically interact with the receptor to form heteromeric assemblies in the cell surface [22, 39–41]. Second, prevalent β-arrestin/ERK1/2 and AKT activation was found at low receptor density [42, 43], while marked cAMP activation would be due to increased G_α_s coupling occurring at relatively high receptor expression levels [22, 23]. These aspects shed light on the physiological impact of extremely variable gonadotropin receptor levels throughout the human menstrual cycle [44]. Agonist binding induces receptor aggregation [45] and sequestration from the cell surface [46], preceding its internalization in endosomal vesicles [47]. In particular, LHCGR internalization is mediated by GPCR kinases and other modulators, such as β-arrestins, that may form super-complexes of signaling modules sustaining prolonged cAMP [48, 49] and direct ERK1/2 activation [23].

Taken together, LHCGR mediates a complex, spatial-temporal network of multiple signaling pathways triggered by LH and hCG. The two hormones act as different ligands linked to specific signaling patterns [3], increasing the complexity of the picture. LH binding to LHCGR leads to preferential activation of proliferative and anti-apoptotic signals delivered through ERK1/2 and AKT, essential to ovarian follicle growth and maturation, while hCG is a potent progestational based on its physiological role [50–54]. These data illustrate the different activity exerted by LH and hCG in vitro, which was confirmed also in a clinical setting [55], and suggest the existence of natural, specific regulatory mechanisms adapting gonadotropin signals to physiological requirements (steroidogenesis, cell death, or survival).

### Knowledge about LH action from in vivo studies

Recent decades have seen the generation of mutant hormone/receptor mice, allowing LH functions to be elucidated in vivo. Results from these studies must be interpreted with caution, since animal models for gonadotropin functioning, especially multi-ovulatory species, cannot be fully representative of human physiology.

One of the first genetically modified mice overexpressed *CGB* and *CGA* [56]. These animals were developed expecting to resemble the phenotype of humans with activating LHCGR mutations (i.e., males affected by testotoxicosis, tumorigenesis, or precocious puberty) and overall asymptomatic females. Instead, transgenic male mice had normal phenotypes, whereas females displayed luteinized ovarian follicles with hemorrhagic cysts and luteomas, precocious puberty, obesity, and other non-reproductive features, such as pituitary adenomas, mammary tumors, and pseudopregnancy [56, 57]. An overall similar phenotype was obtained in female mice overexpressing a chimeric LHβ fused with the carboxyl-terminal peptide of hCG. This molecule has additional glycosylations, extending its half-life, and can lead to polycystic ovaries, relatively high levels of sex steroids, infertility, and ovarian tumors [58].

Knockout mice were developed, starting with the deletion of the *CGA* gene. These animals had obvious hypogonadism and hypothyroidism [59], due to the lack of full LH molecules, as well as FSH and TSH, which share the same α subunit. *LHB* knockout mice were also developed, reflecting this condition in humans. Male mice were affected by hypogonadism and displayed very low androgen levels and hypoplastic Leydig cells despite overall normal FSH levels, while females showed anovulation and collapse of antral follicles, and were infertile [60].

LH receptor knockout mouse models (LuRKO) have phenotypes similar to the LHβ knockout, although they are not fully representative of the condition in humans, where full inactivation of LHCGR leads to type-1 Leydig cell hypoplasia and hermaphroditism. In mice at the fetal stage, the production of testosterone can occur through a gonadotropin-independent pathway [61], stimulating fetal Leydig cells even in the absence of LH or its receptor, given the support of other paracrine factors [62]. Therefore, LuRKO mice display impaired sexual maturation but have similar phenotypes of the wild-type at birth. Interestingly, the effects of the absence of LH receptors may be partially rescued in older mice (≥ 12 months), where LH-independent testosterone production could support qualitatively, but not quantitatively, similar spermatogenesis to wild-type mice [63]. In LuRKO mice, similar effects were obtained by FSH receptor (FSHR) activating mutations, where spermatogenesis is rescued even in the presence of the anti-androgen flutamide [64].

These findings suggest the existence of Sertoli cell–dependent paracrine factors capable of supporting partially Leydig cell functions, reflective of partially overlapping spermatogenic pathways. Moreover, these data suggest that the hormonal regulation of spermatogenesis has shifted from the dominance of gonadotropins to sex steroids during evolution. However, these data were not fully replicated in female LuRKO mice, where the expression of constitutively active FSHR led to the progression of antral follicles to the preovulatory stage and enhanced estrogenic activity but failed to rescue the healthy phenotype from hypogonadism and anovulation [65]. Taken together, studies from in vivo models have confirmed the relevance of the LH/LHCGR system for reproduction, although potential translation of results to humans must take careful account of sex- and species-specific effects.

### The impact of LH and LHCGR genetic variants on signal transduction during folliculogenesis

Few single-nucleotide polymorphisms (SNPs) within the *LHB*/*CGB* gene cluster have been described. In general, though little is known about *CGB* SNPs and their possible association with miscarriage [66], *LHB* SNPs are linked to human phenotypic variations that might mildly contribute to the pathogenesis of reproductive diseases, such as polycystic ovary syndrome (PCOS) [67]. The main molecular mechanism by which *LHB* and *LHCGR* SNPs impact ovarian functions would rely on the modulation of androgen production and, in turn, on the subsequent perturbation of the hypothalamic-pituitary-gonadal axis. However, it is plausible that mitogenic signals mediated directly by gonadotropins, fundamental to support gametogenesis, could also be perturbed. The endocrine adaptation to androgen levels is controlled by feedback mechanisms that lead to changes of serum LH and FSH, as well as levels of gonadal and adrenal steroids. Together, these events impact on gametogenesis and metabolism, as functions that, in large part, are directly or indirectly dependent on gonadotropins and steroids. However, clinical data are limited, and functional characterizations are mostly missing, suggesting that these SNPs do not substantially impact fertility [67].

One of the best-characterized *LHB* polymorphic variants was found in the Finnish population: V-LH, which consists in the double amino acid tryptophan–arginine and isoleucine–threonine change at positions 28 and 35 of the protein chain [68, 69]. It has an additional glycosylation site [70], lower half-life, and receptor binding, as well as decreased potency in activating progesterone and cAMP, than the classical LH form [71, 72]. The V-LH variant was associated with infertility in homozygous Japanese women [73], as well as with the worst outcome of intracytoplasmic sperm injection procedures, reflecting enhanced pro-apoptotic signals detected in vitro, such as caspase 3 cleavage and DNA fragmentation index [74]. However, these data were never extensively replicated by independent studies in different ethnic groups, suggesting that the impact of V-LH on the phenotype is overall weak. This hormone variant is less frequent in obese PCOS women than in non-obese PCOS and healthy women, indicating that it might be protective against certain metabolic features related to the disease [75], although a further investigation failed to confirm this association [76]. Interestingly, the relatively low hormone bioactivity is compensated by higher V-LH expression than LH, due to SNPs falling within the promoter region in linkage disequilibrium with those at positions 25 and 35, increasing its activity in vivo [77, 78]. Together, these data suggest that the V-LH consists of a polymorphic variant associated with overall mild phenotypes.

In women, some other *LHB* SNPs were associated with infertility [79] or central precocious puberty [80]. In particular, a SNP characterized by the synonymous amino acid T–C change within the exon 3 at gene sequence position 294, was found to be more frequent in South Indian women with PCOS compared with healthy controls [81]. Although the role of this SNP in PCOS pathogenesis is unknown, it was hypothesized that it could impact the function of a *LHB* palindromic gene *RUVBL2*, coding a protein involved in the regulation of DNA transcription [82]. Another *LHB* SNP of potential clinical interest is provided by the asparagine–serine change at position 312 (p.Asn312Ser) of the protein chain, which is close to a glycosylation site and could impact sensitivity to the hormone and live birth rate [15, 83, 84]. Although *LHB* polymorphic variants could be promising targets for future pharmacogenomic research, these results require confirmation by independent clinical studies in other populations and functional in vitro support.

Most SNPs modulating LH/hCG signaling are carried by LHCGR [3]. The receptor is a hot-spot for certain reproductive diseases, such as PCOS [85–87], reflecting the relevance of a fine-tuned regulation of LH signaling to support folliculogenesis. Although the mechanism of PCOS pathogenesis is still largely unclear, marked LHCGR-dependent signals are likely important. They could lead to excessive androgen production which, in turn, impacts the endocrine control of the hypothalamus-pituitary-gonadal axis, interfering with ovarian follicular maturation and metabolism [88]. In fact, several *LHCGR* SNPs were associated with PCOS in different populations [89–96]. For instance, the exon 10 SNP characterized by the alanine–serine change at position 312 was associated with serum LH levels in PCOS patients [97]. Interestingly, the same SNP was linked to spermatogenic damage and infertility in males [98], suggesting that the variant falls within a key region for receptor functioning. Beyond PCOS, the possible link between *LHCGR* SNPs and clinical outcome of assisted reproduction, such as oocyte/embryo quality, was also discussed [99]. Although these findings are encouraging, the LHCGR-dependent molecular mechanism at the basis of PCOS pathogenesis remains poorly understood and the role of the receptor as a potential target for pharmacological approach to the disease is under-researched [99]. The clinical effect of LHβ and LHCGR genetic variants, as well as of possible pharmacogenomic approaches, will be discussed in the next sections.

### The clinical effect of *LHB* genetic variants

In COS, it has been suggested that elevated requirement of FSH may result from weak LHCGR activity. Therefore, these patients might benefit from exogenous LH administration rather than increased FSH dose. This effect could be linked to V-LH which, in the context of COS, cannot achieve adequate levels to compensate for its reduced bioactivity. In a retrospective analysis [100], where patients were divided into three groups according to the FSH dose required, the frequency of V-LH was higher in women with ovarian resistance to FSH administration in association with a lower number of oocytes retrieved. The LH versus V-LH genotypes were stratified in another multicentric study, demonstrating that elevated cumulative doses of FSH were associated with the V-LH genetic variant [16]. The mean number of oocytes retrieved, fertilization rate, and pregnancy rate did not differ between the two groups, indicating that the highest doses of FSH may counterbalance the negative impact of low V-LH bioactivity in inducing oocyte competence and impacting on IVF outcome. However, a significant reduction in the mean of embryo number transferred has been reported. The investigators posited an interplay between FSH- and LH-mediated signals to determine successful oocyte maturation and meiosis [16]. Recently, V-LH was associated with a lower pregnancy rate in gonadotropin-releasing hormone (GnRH) antagonists, but not in long GnRH agonist cycles; these differences were attributed to variations in clinical protocols [101]. Endogenous LH levels were lower with the antagonist cycle, compared with the agonist cycle [102–104]. Therefore, V-LH carriers are associated with reduced pregnancy rates only when they undergo profound LH suppression induced by antagonist protocols.

Another clinically interesting LHβ variant consists in the single missense exon 3 variation, consisting in the amino acid serine–glycine changes at position 102 (Gly1502Ser, rs1056917). This variant may change LH bioactivity, since a single study reported the association between the SNP and reduced LH level, and history of infertility [105]. However, another study prospectively enrolling 220 women undergoing long protocol for COS and IVF found no significant association between this SNP and ovarian response [106], suggesting that overall effects of this genetic variation are likely mild.

### The clinical effect of LHCGR genetic variants

#### LHCGR exon 10 polymorphisms

One of the most studied polymorphisms is the exon 10 p.Asn312Ser. It is relatively common: in European Caucasians, the allele frequency is 41% asparagine (Asn) and 49% serine (Ser), compared with 68% Asn and 32% Ser in the Sub-Saharan African population (https://www.ncbi.nlm.nih.gov/snp/rs2293275). It was demonstrated that Asn-homozygous women required a lower amount of gonadotropin per day, during COS, than Ser-homozygous [84], while the latter have a fourfold higher chance of pregnancy than Asn-homozygous. In the same population, authors later reported significantly higher live birth and cumulative live birth rates in Ser-variant versus Asn-variant carriers [83]. This was explained by the increased number of good-quality embryos found in the Ser carrier group [83], according to the guidelines by Gardner and Schoolcraft [107, 108]. Consistently with Lindgren findings, in a prospective study involving 210 women, higher clinical pregnancy rate was observed in Ser-homozygous carriers than heterozygous women after fresh embryo transfer [109]. However, we obtained different results in a multicenter retrospective study involving 94 normogonadotropic women from three European IVF centers, where no significant association was found in terms of ovarian response (number of oocytes retrieved, MII oocytes) and pregnancy rate among different LHCGR haplotypes [110]. Our findings were corroborated by a recent analysis involving 1183 women, ages 18–40 years and undergoing their first assisted reproductive technology cycle, where the association between the LHCGR Asn312Ser variant and pregnancy rate was not detected [111]. Discrepancies between those studies could be linked to differences in their study designs, IVF protocols adopted, and ethnicity of participants.

Another exon 10 LHCGR polymorphism consists of the Asn–Ser change at position 291 (rs12470652). This variant was associated with increased receptor sensitivity [112] and has a prevalence of 5% in Europe (https://www.ncbi.nlm.nih.gov/snp/rs12470652). The clinical relevance of this polymorphism was assessed; no association was found with PCOS risk [113, 114], nor response to testicular cancer treatment [115]. In our multicenter prospective studies, we observed that this variant was associated with greater response to COS, evaluated as oocytes, and mature oocytes retrieved [110, 116]. In combination with other FSHR polymorphisms, this variant is associated with the ratio between cumulative FSH dose and total number of oocytes retrieved (odds ratio [OR] 5.44, 95% CI 3.18–7.71; *p* < 0.001), supporting the concept of varying sensitivity to the ligand depending on LHCGR Asn291Ser phenotype [110, 116]. These results were confirmed by recent research demonstrating that the Ser variant was significantly associated with PCOS risk, which is typically characterized by an increased sensitivity to exogenous gonadotropin during COS. Unfortunately, the evidence reported so far is limited due to the overall low prevalence of the Ser variant and a paucity of data on homozygotic carriers [113, 114, 116].

#### LHCGR A–G intronic nucleotide variation

Another LHCGR polymorphism consists in the A–G intronic nucleotide variation (rs13405728), which was strongly associated with PCOS and first described in a genome-wide association study in Chinese women [85]. The G allele occurs in 8% of the global population, with the highest prevalence in Africa (27–31%); https://www.ncbi.nlm.nih.gov/snp/rs13405728#frequency_tab). A meta-analysis revealed that the OR for developing PCOS was significantly lower in G than in A carriers (OR 0.735, 95% CI 0.699–0.773; *p* < 0.001). Consistent with these results, a recent case–control study involving 400 PCOS women compared with 480 healthy controls confirmed the reduced risk among G carriers of developing PCOS syndrome [117]. The mechanism by which this polymorphism could promote PCOS is still not understood. A recent study suggested that *LHCGR* rs13405728 could modulate the *STON1* and *FSHR* transduction, thereby influencing metabolic processes and androgen receptor expression [118].

#### LHCGR missense polymorphisms

Finally, LHCGR missense polymorphism Asn312Ser (rs2293275) has been found to be associated with PCOS [93, 113, 119, 120]. In a meta-analysis collecting six association studies, Caucasian homozygous Ser carriers have an increased risk of developing ovarian hyperstimulation syndrome (OHSS) than Asn-homozygous and heterozygous (OR 4.11, CI 95% 1.03–16.38) [119]. Recently, a study involving 421 PCOS and 322 regularly menstruating women found the highest prevalence of the Ser variant among individuals with the disease [91].

### Pharmacogenomic approach in women with clinical variants affecting the LH system

The pharmacogenomic approach consists of the prescription of medication based on the individual genetic profile. Specific genetic variants could influence the pharmacokinetics or pharmacodynamics of a drug. For instance, some individuals have increased or reduced receptor sensitivity to an exogenous medication or display a different drug half-life. In patients at risk of adverse events, the pharmacogenomic approach could help to minimize safety issues [121]. In the context of assisted reproduction, we have at least two possible adverse events linked to exogenous gonadotropin administration. One is OHSS, potentially a life-threatening event, in which an exaggerated ovarian response to gonadotropins occurs, leading to the development of multiple follicles, ascites, and thrombotic events [122, 123]. Moreover, women could have poor ovarian response to gonadotropins, leading to poor outcomes with assisted reproduction techniques and dropout from IVF treatment [124].

So far, very few studies have been conducted using a pharmacogenomic approach in the context of IVF. Regarding SNPs modulating LH/LHCGR signals, a prospective analysis of 193 women with a history of unsuccessful IVF cycles was performed [14]. Seventy-eight women were supplemented with 75 UI of LH from day 6 (control group), while 115 were supplemented with LH, according to LHCGR Asn312Ser phenotype, from day 1 (study group). Homozygous Asn carriers received no LH; 37.5 UI of LH was prescribed to heterozygous, and 75 UI to Ser-homozygous, carriers. Women receiving genotype-based LH personalized treatment had a higher clinical pregnancy rate than the control group (56/115 vs. 26/78; *p* = 0.049). However, limitations of this study must be acknowledged, such as its retrospective design and the duration of LH treatment, which was significantly higher in the study group than in controls. In another retrospective analysis, 533 women underwent a long protocol with FSH and LH coadministration, according to previously established criteria [125]. Authors found that Ser-homozygous women required more LH during COS, although they had a higher rate of pregnancies than women with other haplotypes [15].

## Knowledge gaps and future research

Pharmacogenomics aims to improve the efficacy of therapeutic approaches, based on the susceptibility of genetic profiles. However, what is still debated is whether to promote the widespread use of this genetic information in clinical practice, since only a limited set of genetic variations have significant impact.

In specific subsets of ART patients, the reproductive outcome may benefit from LH supplementation. These patients consist in women with hypo-response to exogenous FSH alone [126–129], and women of advanced reproductive age (≥ 35 years) [130]. The genetic aspects beyond LH deficiency have been poorly understood. As previously reported, serine carriers of the LHCGR variant (rs2293275) may require higher amounts of LH, as a FSH supplement during COS, than other LHCGR genotypes [15]. Moreover carriers of LHB variant might need increased FSH dosage during COS [100]. The impact of LHCGR variant on pregnancy and cumulative pregnancy was observed by just one research group [83, 84] but unfortunately not corroborated by other studies [110, 111]. These mixed findings could be explained by inter-study differences in design and protocols. Another issue concerning pregnancy rate resides in the difficulty to reach the adequate sample size in IVF studies [131]. Finally, in our opinion the interaction among polymorphisms is still under-investigated. In other words, instead of focusing on single genetic association, future studies should focus more on simultaneous analysis of the genetic variants involved in COS [110, 132, 133].

So far, the lack of studies on gonadotropin polymorphisms could be explained by high costs of genetic tests, in comparison to hormonal assays, which are easier to perform, less expensive, and routinely used in clinical practice to guide COS during ART.

Dose-finding procedures, appropriate protocols, duration of stimulation, and number of cycles all warrant further investigation to improve our knowledge of ovarian response. Hence, the identification of genetic determinants for reduced ovarian response might lead to tailored medical approaches, reduce the overall costs of IVF treatments, and improve COS efficiency.

Predictive medicine also takes advantage of indexes to assess specific conditions. In ART, parameters such as the Follicle to Oocyte Index (FOI) and Follicular Output Rate (FORT) have been applied to practically define the ovarian response to COS [134]. However, no studies have been performed to evaluate the association between specific gonadotropin polymorphisms and ovarian sensitivity indexes. This knowledge might play a key role in increasing the chance of recruiting more oocytes and optimizing the chance of a live birth [135]. Informative results on whether such genetic polymorphisms could be associated with reduced response to COS may be obtained from poor responder women, who belong to groups 3 and 4 of the POSEIDON classification. Nonetheless, there is a need for large multicenter studies of real-world data, across different ages and ethnic groups, to further evaluate benefits of genetic testing in ART.

## Conclusion

The pharmacogenomic approach based on *LHB* and/or *LHCGR* genotypes remains under-researched. The most promising SNP that might be used to personalize COS is the LHCGR Asn312Ser, but the evidence so far is poor and limited to very few studies. Although a possible cumulative effect between LH and LHCGR SNPs has been reported in several studies, these findings have yet to be supported by any randomized clinical trials. Without such investigations, it is not yet possible to suggest a pharmacogenomic approach in clinical practice.

## Supplementary Information

Supplementary Material 1.

## Data Availability

Not applicable.

## Abbreviations

AKT: Protein kinase B
AMP: Adenosine monophosphate
ART: Assisted reproductive therapy
Ca^2+^: Calcium ion
cAMP: Cyclic adenosine monophosphate
CGA: Human α subunit-coding gene
CGB: Human β subunit-coding gene
COS: Controlled ovarian stimulation
CREB: cAMP response element–binding protein
ERK1/2: Extracellular-regulated kinase 1/2
FSH[R]: Follicle-stimulating hormone [receptor
GnRH: Gonadotropin-releasing hormone
GPCR: G protein-coupled receptor
hCG: Human chorionic gonadotropin
IP_3_: Inositol trisphosphate
IVF: in vitro fertilization
LH: Luteinizing hormone
LHCGR: Luteinizing hormone/choriogonadotropin receptor
LuRKO: LH receptor knockout
OHSS: Ovarian hyperstimulation syndrome
OR: Odds ratio
PCOS: Polycystic ovary syndrome
PKA: Protein kinase A
PLC: Phospholipase C
SNP: Single-nucleotide polymorphism
TSH: Thyroid-stimulating hormone
