# Supplementary material for: Pharmacogenomic of LH and its receptor: are we ready for clinical practice?
Source: Reprod Biol Endocrinol. 2025 Feb 25;23(Suppl 1):29. doi: 10.1186/s12958-025-01359-2 (PMC11863420; doi:10.1186/s12958-025-01359-2)
Supplement: Supplementary file 1 — Supplementary Material 1. [file 12958_2025_1359_MOESM1_ESM.docx]

| **Author, year** | **Sample size** | **Aim** | **Genetic variants of LH gene** | **Genetic variants of LHCGR** | **Main findings** |
| --- | --- | --- | --- | --- | --- |
| Alviggi et al., 2011 | 60 undergoing IVF | To estimate the association between v-βLH and the profile of ovarian response to recombinant human FSH (rhFSH). | V-LHβ Trp8Arg and Ile15Thr  (rs1800447/ rs34349826) | - | v-betaLH is more frequent in women with ovarian resistance to rhFSH. |
| Alviggi et al., 2013 | 220 women undergoing IVF | To confirm that women with V-LHβ display hypo-sensitivity to exogenous FSH in a Danish female population. | V-LHβ Trp8Arg and Ile15Thr  (rs1800447/ rs34349826) | - | v-betaLH is significantly associated wht higher cumulative-dose of r-hFSH |
| Davar et al., 2014 | 220 women undergoing IVF | To evaluate the accurate prognosis of COH by evaluating the correlation with polymorphisms | V-LHβ Gly102Ser  (rs14957983) | - | no significant relationship between this polymorphism and the ovarian response. |
| Lindgren et al., 2016 | 384 women undergoing IVF | To investigate if described polymorphisms in the FSHR and LHCGR genesimpact IVF outcomes and clinical parameters in IVF trial | - | LHCGR N312S  (rs2293275) | Women homozygous for serine in both polymorphisms displayed higher pregnancy rates than women homozygous asparagine |
| Ramaraju et al., 2018 | 553 women undergoing IVF | To investigate the effect of LHCGR N312S polymorphism in determining the need of r-hLH supplementation with r-hFSH in patients undergoing ART. | - | LHCGR  N312S  (rs2293275) | Women heterozygous and homozygous for S allele required higher doses of r-hLH and have higher clinical pregnancy rates. |
| Lindgren et al., 2019 | 665 women undergoing IVF | To investigate if the gonadotropin receptor variants N680S (N: asparagine, S: serine, rs6166) in the FSHR and N312S (rs2293275) in the LHCGR predict CLBR | - | LHCGR  N312S  (rs2293275) | Women homozygous for S in both receptors combined (4S) had significantly higher live birth rate compared to those with other receptor variants in the first three IVF cycle |
| Ramaraju et al., 2021 | 193 patients in a second IVF cycle | To evaluate the impact of a personalized pharmacogenomic approach on LH supplementation on the pregnancy and live birth rate outcomes |  | LHCGR-N312S  (rs2293275) | Significant increase in pregnancy rate and improvement in live birth rates when r-hLH supplementation protocol was decided as per the genotypes N/N, N/S, and S/S of the N312S variant in the respective patients |
| Ku et al., 2021 | 591 women undergoing IVF | To clarify the effect of COH protocols, IVF outcomes in the GnRH antagonist and long GnRH agonist protocol groups | V-LHβ Trp8Arg and Ile15Thr | - | The variant LH beta gene was associated with a lower clinical pregnancy rate in GnRH antagonist cycles but not in long GnRH agonist cycles. |
| Guo et al., 2021 | 210 women undergoing IVF | To study the association between | - | LHCGR  N312S  (rs2293275) | The LHCGR N321S variant is associated with increased clinical pregnancy rate of fresh embryo transfer in infertile women |
| Pirtea et al., 2022 | 1,183 women undergoing IVF | To study consequences of LHCGR SNPs on assisted reproductive technology outcomes | - | LHCGR-N312S  (rs2293275) | No association with assisted reproductive technology outcome |
| Alviggi et al., 2023 | 94 normogo- nadotropic IVF/ICSI patients came from three public IVF Units. | To evaluate the effect of polymorphisms of gonadotropins and their re ceptors on ovarian stimulation outcomes in IVF patients co-treated with a GnRHa long down-regulation protocol | - | LHCGR  N291S  (rs12470652)  LHCGR-N312S  (rs2293275) | Basal estradiol levels, number of fertilized and mature oocytes were lower in homozygotic carriers of LHCGR-291 (N/N) compared to heterozygotic N/S |

AS-qPCR, allele-specific real-time quantitative PCR; COS, controlled ovarian stimulation; CLBR, cumulative live birth rate after in vitro fertilization; IVF, in vitro fertilization; HRM, high resolution melting; HPG-axis, hypogonadotropic-axis; FSHR, follicular stimulation receptor; LHCGR, lutropin receptor; LHβ, luteinizing hormone beta subunit; LH, luteinizing hormone; LHB, luteinizing hormone beta gene; LHβ W8R and I15T: V- LHβ variant; LHCGR, luteinizing hormone/choriogonadotropin receptor; PCOS, polycystic ovary syndrome; SNP, single-nucleotide polymorphism; SNVs, single nucleotide variants.
